# The role of site-2-proteases in bacteria: a review on physiology, virulence, and therapeutic potential

**DOI:** 10.1093/femsml/uqad025

**Published:** 2023-05-03

**Authors:** Sofie S Kristensen, Dzung B Diep, Morten Kjos, Geir Mathiesen

**Affiliations:** Faculty of Chemistry, Biotechnology, and Food Science, Norwegian University of Life Sciences (NMBU), 1433 Ås, Norway; Faculty of Chemistry, Biotechnology, and Food Science, Norwegian University of Life Sciences (NMBU), 1433 Ås, Norway; Faculty of Chemistry, Biotechnology, and Food Science, Norwegian University of Life Sciences (NMBU), 1433 Ås, Norway; Faculty of Chemistry, Biotechnology, and Food Science, Norwegian University of Life Sciences (NMBU), 1433 Ås, Norway

## Abstract

Site-2-proteases are a class of intramembrane proteases involved in regulated intramembrane proteolysis. Regulated intramembrane proteolysis is a highly conserved signaling mechanism that commonly involves sequential digestion of an anti-sigma factor by a site-1- and site-2-protease in response to external stimuli, resulting in an adaptive transcriptional response. Variation of this signaling cascade continues to emerge as the role of site-2-proteases in bacteria continues to be explored. Site-2-proteases are highly conserved among bacteria and play a key role in multiple processes, including iron uptake, stress response, and pheromone production. Additionally, an increasing number of site-2-proteases have been found to play a pivotal role in the virulence properties of multiple human pathogens, such as alginate production in *Pseudomonas aeruginosa*, toxin production in *Vibrio cholerae*, resistance to lysozyme in enterococci and antimicrobials in several *Bacillus* spp, and cell-envelope lipid composition in *Mycobacterium tuberculosis*. The prominent role of site-2-proteases in bacterial pathogenicity highlights the potential of site-2-proteases as novel targets for therapeutic intervention. In this review, we summarize the role of site-2-proteases in bacterial physiology and virulence, as well as evaluate the therapeutic potential of site-2-proteases.

## Introduction

Bacteria depend on transmembrane signaling to sense and quickly respond to a changing environment. Fast response is particularly crucial for pathogenic bacteria, which encounter rapidly changing environment within the host throughout the infection process. Regulated intramembrane proteolysis (RIP) is a signaling mechanism known to mediate transmembrane signaling in Gram-positive and Gram-negative bacteria (Urban 2009, Schneider and Glickman 2013, Wettstadt and Llamas 2020). RIP is mediated by a unique class of intramembrane proteases (IMP). IMP are divided into four families based on their catalytic mechanisms: The rhomboid proteases (serine proteases), the site-2-metalloproteases (S2P; zinc metalloproteases), Rec1 (glutamyl protease) and the presenilin/signaling peptide peptidases (aspartyl proteases). While the catalytic mechanism varies between the different IMP families, structural determination of prototypic members has confirmed the membrane-embedded location of the catalytic active site for each family (Wu et al. 2006, Feng et al. 2007, Li et al. 2013, Manolaridis et al. 2013, Bai et al. 2015). All members are proposed to contain a hydrophilic cavity or channel, delivering water from either side of the membrane to the active site, thereby allowing hydrolysis of peptide bonds within the cell membrane (Wu et al. 2006, Feng et al. 2007, Li et al. 2013, Manolaridis et al. 2013, Bai et al. 2015). Such intramembrane hydrolysis of the peptide substrate is often a critical step in cell signaling. In the present review, the role of S2P-mediated RIP signaling in bacteria will be discussed. The remaining IMP families have been extensively reviewed in other articles (Wolfe and Kopan 2004, Urban 2009, Sun et al. 2016, Verhelst 2017, Beard et al. 2019).

**Figure 1.**
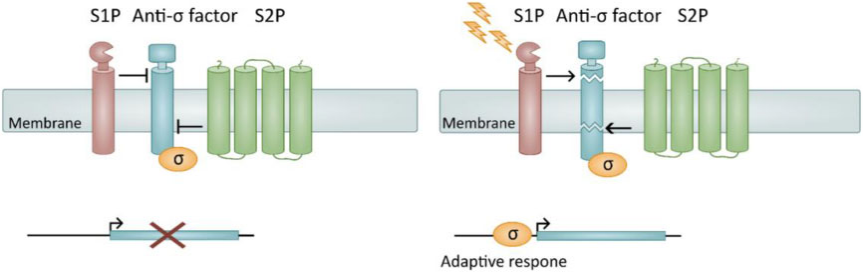
The general S2P-mediated RIP paradigm. In the absence of environmental cues (left panel), the cleavage of the anti-sigma factor is inhibited, leaving the bound σ-factor in an inactive state. When extracytoplasmic stimuli are detected (right panel), a site-1-protease (S1P) cleaves a membrane-bound anti-sigma factor on the periplasmic side. The primary cleavage triggers a secondary cleavage by a site-2-protease (S2P), resulting in release of the σ-factor (orange) from the membrane, and activation of genes involved in adaptive response.

**Figure 2.**
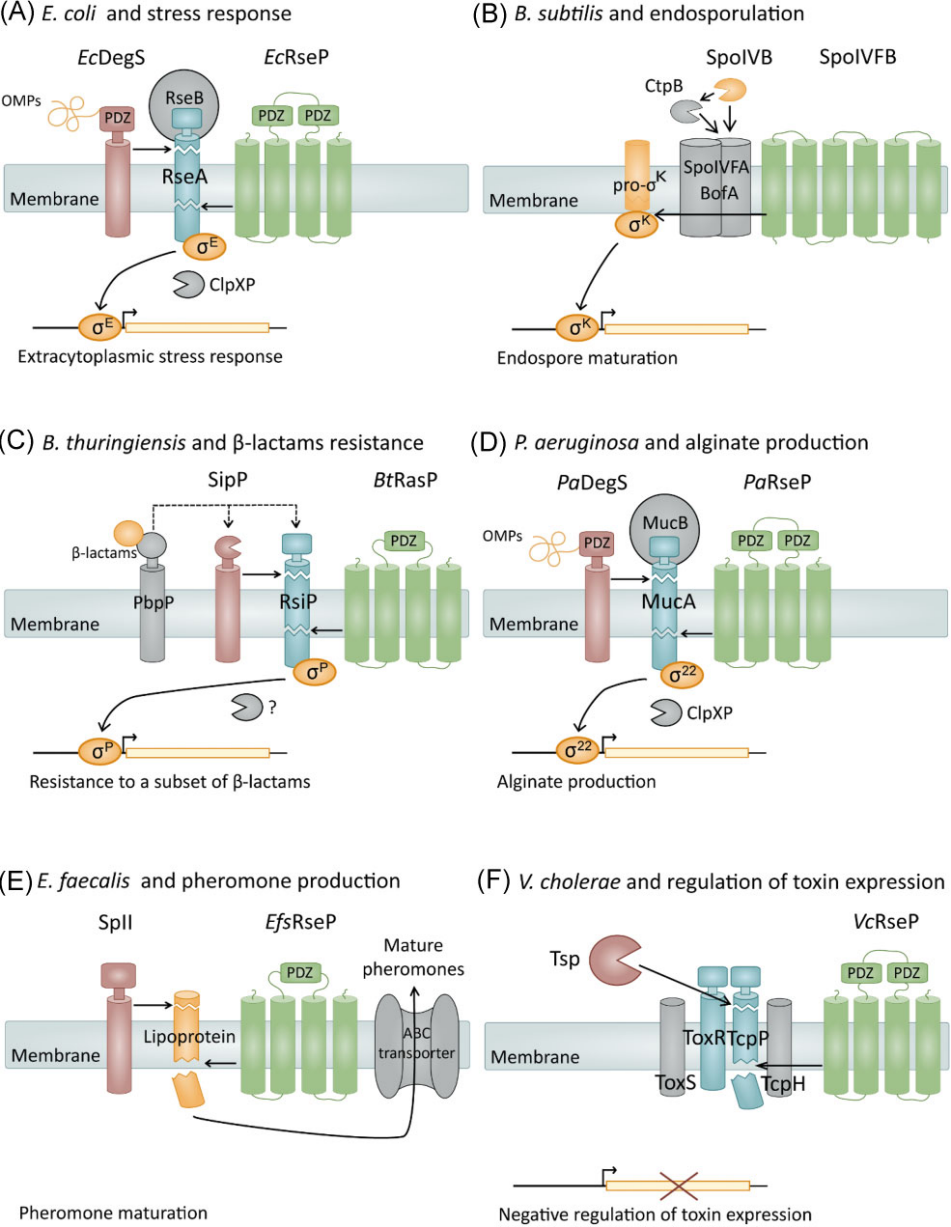
The role of S2P in RIP signaling mechanism in selected bacteria. (A) The *Escherichia coli* RseP activation of the extracytoplasmic stress response serves as a blueprint for S2P-mediated signaling. Accumulation of unfolded outer membrane proteins (OMPs) is a known stress cue sensed by the PDZ domain of the S1P DegS. The accumulation of OMPs results in sequential cleavage of the anti-sigma factor RseA by DegS (site-1-cleavage) and RseP (site-2-cleavage). The RseP cut reveals a degron in the anti-sigma domain which is subsequently cleaved by ClpXP in the cytosol. The resulting release of σ^E^ activates genes involved in the adaptive stress response. RseB, a periplasmic protein, negatively regulates σ^E^ activity by blocking DegS/RseP cleavage under non-stress conditions. (B–F) Variations of RIP involving S2P are shown for various species. In all panels: The suggested stimuli signal is shown in yellow, the substrate in blue, presumed S1P in red, S2P in green, sigma factors (A–D) or lipoprotein (E) in active form in orange, and additional components in grey. See the text for more details.

In bacteria, gene expression is commonly controlled at the transcriptional level. S2P-mediated RIP signaling allow regulated gene expression in bacteria by activating an alternative sigma factor of the extracytoplasmic function (ECF) family in response to environmental cues. As reviewed in detail here and by others, S2P-mediated RIP signaling contributes to a vast array of cellular processes, including cell polarity in *Caulobacter crescentus*, iron acquisition in various Gram-negative bacteria and sporulation in *Bacillus subtilis* (Chen et al. 2005, King-Lyons et al. 2007, Yokoyama et al. 2021). Most notably, RIP-mediated activation of sigma factors has been linked to virulence in several human pathogens (Schöbel et al. 2004, Makinoshima and Glickman 2006, Urban 2009, Schneider and Glickman 2013). While regulating a vast array of cellular processes, S2P-mediated RIP signaling generally adheres to the same general principle (Fig. 1) (Chen and Zhang 2010). A membrane-bound anti-sigma factor, commonly a single-spanning membrane protein sequesters the ECF sigma factor to the membrane, thereby negatively regulating ECF sigma factor activity. Following the detection of an environmental signal, a primary site-1-protease (S1P) cleaves the anti-sigma factor (site-1-cleavage). This primary cleavage triggers an intramembrane protease (IMP) to cleave the anti-sigma factor within the lipid bilayer (site-2-cleave), thereby releasing the active σ-factor from the membrane. The RIP cascade is a widely distributed signaling pathway in the bacterial world, and variations of this blueprint are emerging as the role of S2P in different bacteria are explored (Fig. 2, Table 1).

**Table 1.** Suggested components of the RIP signaling pathway in selected bacteria.

Organism	S1P	Substrate	S2P	Transcr. regulator	Function	Ref.
*Bordetella bronchiseptica*	‡	HurR	HurP	σ^HurI^	Iron acquisition	(King-Lyons et al. 2007)
*B. subtilis*	n.r.	Pro-σ^K^	SpoIVFB	σ^K^	Sporulation	(Rudner et al. 1999, Yu and Kroos 2000, Campo and Rudner 2006)
	SipS SipT	RsiV	RasP	σ^V^	Lysozyme resistance	(Hastie et al. 2013, Castro et al. 2018)
	PrsW	RsiW	RasP	σ^W^	Cell envlope stress response	(Cao et al. 2002, Schöbel et al. 2004, Ellermeier and Losick 2006, Heinrich and Wiegert 2006)
*Bacillus thuringiensis*	SipP	RsiP	RasP	σ^P^	Resistance to a subset of β-lactams	(Ho et al. 2019, Nauta et al. 2022)
*C. crescentus*	PerP	PodJ_S_	MmpA	None	Cell polarity	(Chen et al. 2005, Chen et al. 2006)
*Clostridioides difficile*	‡	RsiV	RasP	σ^V^	Lysozyme resistance	(Pannullo and Ellermeier 2022)
*E. coli*	DegS	RseA	RseP	σ^E^	Cell envelope stress response	(Alba et al. 2002)
	‡	FecR	RseP	σ^FecI^	Iron acquisition	(Yokoyama et al. 2021)
*Enterococcus faecalis*	‡	RsiV	RseP	σ^v^	Lysozyme resistance	(Varahan et al. 2013)
	SPII	Signal peptides	RseP	None	Pheromone production	(An et al. 1999, An and Clewell 2002, Antiporta and Dunny 2002, Chandler et al. 2005)
*Listeria monocytogenes*	‡	Signal peptides	Eep	None	Pheromone production	(Xayarath et al. 2015)
*Mycobacterium tuberculosis*	‡	RslA,	Rip1	σ^L^	Regulation of membrane composition, virulence, stress	(Sklar et al. 2010)
	‡	RsmA,	Rip1	σ^M^		(Sklar et al. 2010)
	‡	RskA	Rip1	σ^K^		(Sklar et al. 2010)
	‡	RsdA	Rip1	σ^D^		(Schneider et al. 2014)
*Pseudomonas aeruginosa*	‡	FpvR	RseP	σ^FpvI^ σ^PvdS^	Iron acquisition	(Draper et al. 2011)
	‡	FoxR	RseP	σ^FoxI^		(Draper et al. 2011)
	‡	FiuR	RseP	σ^FiuI^		(Draper et al. 2011, Bastiaansen et al. 2014)
	‡	HasS	RseP	σ^HasI^		(Otero‐Asman et al. 2019)
	‡	HxuR	RseP	σ^HxuI^		(Otero‐Asman et al. 2019)
	DegS	MucA	RseP	σ^22^	Alginate production	(Damron and Goldberg 2012)
*Pseudomonas putida*	Prc	LutY	RseP	σ^IutY^	Iron acquisition	(Bastiaansen et al. 2014)
*Staphylococcus aureus*	‡	Signal peptides	Eep	None	Peptide maturation, Virulence	(Cheng et al. 2020, Schilcher et al. 2020)
*Streptococcus*	‡	Signal peptides	Eep	None	Signal peptide production	(Denham et al. 2008, Chang et al. 2011, Pérez-Pascual et al. 2015)
*Vibrio cholerae*	TsP	TcpP	RseP	TcpP	Downregulates toxin production	(Matson and DiRita 2005, Teoh et al. 2015)
	‡	ToxR	RseP	ToxR	Downregulates toxin production	(Matson and DiRita 2005)

‡, unknown; n.r., not required.

All S2P contain a catalytic core region consisting of three transmembrane segments, with the conserved zinc-binding motif HExxH and the zinc-coordinating region of the LDG motif located on separate transmembrane segments. The conserved glutamate residue of the HExxH motif is suggested to activate a water molecule bound to the zinc, thereby initiating the nucleophilic attack on the peptide bond. Mutations in the conserved catalytic motifs (HExxH, LDG) has been associated with reduced protease activity of S2P in multiple species (Feng et al. 2007, Koide et al. 2007, Saito et al. 2011). Based on phylogenetic analysis, the S2P family can be divided into four subgroups (Kinch et al. 2006). Group I, the largest subgroup, is characterized by a variable number of PDZ domains (IPR001478, structural domain commonly found in signaling proteins). Group III contains a cystathionine β-synthase (CBS) domain (IPR000644, structural domain commonly found in a variety of proteins), but lacks the PDZ domain. With the exception of the S2P of *B. subtilis* and *Methanocaldococcus jannaschii*, all S2P discussed in this review belongs to subgroup I. The two remaining subgroups (Group II and IV), lacks any additional domains; however, none of these putative proteases have been extensively characterized (Kinch et al. 2006). For more details on the biochemical and structural features of S2P, the reader is directed to other reviews (Kroos and Akiyama 2013, Sun et al. 2016).

In this review, the roles and mechanisms of RIP and S2P in bacteria will be explored, starting with the implications of S2P in bacterial adaptation to a rapidly changing environment. Next, RIP-mediated virulence will be reviewed, focusing on the role of S2P in important human pathogens. Finally, we will discuss the therapeutic potential of S2P and highlight the challenges and opportunities of targeting this unique class of proteins.

## The role of S2P in response to changes in the extracytoplasmic environment

Bacteria depend on signal transduction across the membrane to respond appropriately to environmental cues. Proteolysis is a rapid process, thus, multiple signaling pathways depend on proteases to generate an immediate response (Wettstadt and Llamas 2020). Following the discovery of the S2P RseP in *E*. coli and the S2P SpoIVFB in *B. subtilis* at the beginning of this millennium, several S2P have been implied in adaptive response (Table 1, Rudner et al. 1999, Yu and Kroos 2000, Kanehara et al. 2001) (Fig. 2). The following section will discuss the role of S2P in response to changes in the extracytoplasmic environment.

## The role of RseP in regulation of the σ^E^-dependent stress response in *E. coli*

*Escherichia coli* RseP (Regulator of sigma E, protease; *Ec*RseP), previously known as Yael, is one of the most extensively studied members of the S2P family and was first identified as a key regulator of the σ^E^-dependent stress response (Fig. 2A) (Kanehara et al. 2001, Alba et al. 2002, Kanehara et al. 2002). In *E. coli*, the extracytoplasmic stress response is initiated following the sequential digestion of the anti-sigma factor RseA by the S1P DegS and the S2P *Ec*RseP (Ades et al. 1999, Alba et al. 2002, Kanehara et al. 2002). *Ec*RseP-mediated cutting of RseA is one of the best understood transmembrane signaling pathways involving a S2P and is viewed as the blueprint for the bacterial RIP-cascades.

In the absence of stress, σ^E^ is sequestered in the membrane by the anti-sigma factor RseA (De Las Peñas et al. 1997, Missiakas et al. 1997) (Fig. 2A). During stress conditions, such as heat, unfolded or misfolded OMPs will accumulate in the periplasm. Unfolding of OMPs results in exposure of hydrophobic amino acids that is directly recognized by the periplasmic PDZ domain of the S1P DegS (Walsh et al. 2003). Recognition of unfolded OMPs triggers DegS-mediated cleavage of the periplasmic region of RseA, which subsequently initiates a secondary cleavage by the S2P *Ec*RseP (Ades et al. 1999, Alba et al. 2002, Kanehara et al. 2002, Walsh et al. 2003, Akiyama et al. 2004). *Ec*RseP cleaves RseA within the transmembrane segment, thereby releasing the cytoplasmic region of RseA in complex with σ^E^ (Akiyama et al. 2004, Li et al. 2009). The protease ClpXP performs the final cleavage of RseA to release the σ^E^ into the cytosol, which activates the transcription of target genes involved in the stress response (Flynn et al. 2004).

The RIP-mediated activation of σ^E^ is tightly regulated at multiple levels to ensure that σ^E^ activation is strictly stress-dependent (Fig. 2A). Most importantly, *Ec*RseP can only efficiently process RseA following site-1-cleavage (Li et al. 2009). Many proteins involved in RIP, including both DegS and *Ec*RseP, harbors extracellular PDZ domains, which are thought to be important for structuring of signal complexes. It has been suggested that the *Ec*RseP PDZ domain acts as a size-exclusion filter, preventing interaction with larger substrates such as full-length RseA (Hizukuri et al. 2014). Two lines of evidence support this notion. First, mutational analysis has demonstrated that disruption of the *Ec*RseP PDZ domain results in *Ec*RseP-mediated cleavage of RseA in the absence of site-1-cleavage (Kanehara et al. 2003, Bohn et al. 2004, Inaba et al. 2008). Second, structural analysis indicates that the PDZ tandem forms a pocket-like structure facing the catalytic center (Hizukuri et al. 2014, Imaizumi et al. 2022), thereby blocking the access of bulky substrates to the active site. Notably, the newly exposed C-terminal of DegS-cleavage RseA is suggested to interact directly with the PDZ domain (Li et al. 2009), further highlighting the importance of this domain in substrate discrimination. In addition, the periplasmic stress sensor protein RseB function as a negative regulator of σ^E^ activity. RseB interacts with the periplasmic region of RseA, presumably inhibiting DegS cleavage by masking the site-1-cleavage site (De Las Peñas et al. 1997, Missiakas et al. 1997, Cezairliyan and Sauer 2007, Kim et al. 2010). During stress, misfolded OMPs and lipoprotein intermediates are suggested to promote RseB release from RseA (Wollmann and Zeth 2007, Chaba et al. 2011, Lima et al. 2013). These regulation mechanisms collectively ensure that *Ec*RseP-mediated activation of σ^E^ is strictly stress-dependent.

## SpoIVFB and endospore formation in *B. subtilis*

SpoIVFB (Stage IV sporulation protein FB), the S2P of *B. subtilis* controlling the transcription factor σ^K^, is another well-characterized member of the S2P family (Fig. 2B) (Rudner et al. 1999, Yu and Kroos 2000, Zhou et al. 2009). Under harsh environmental conditions, actively growing *B. subtilis* differentiates into a dormant state, known as endospores, one of the most advanced and long-lasting forms of stress response found among bacteria (Tan and Ramamurthi 2014). The regulatory mechanisms of endospore formation are complex, involving a set of sigma factors which are active at specific time points during sporulation. The transcription factor σ^K^ controls the final stage of the endospore formation. σ^K^ is synthesized as an inactive precursor, pro-σ^K^, which is sequestered in the outer forespore membrane (Zhang et al. 1998). SpoIVFB cleaves the N-terminal pro-region of pro-σ^K^, resulting in mature σ^K^ being released into the mother cell (Rudner et al. 1999, Zhou et al. 2009). Once released, σ^K^ activates genes needed in the final stage of endospore maturation (Fig. 2B).

Analogous to *Ec*RseP cleavage of RseA (Fig. 2A), the SpoIVFB cleavage of pro-σ^K^ is tightly regulated (Fig. 2B), to make sure that σ^K^ is activated only at the correct developmental stage of sporulation. As discussed above, regulation of *Ec*RseP activity largely relies on substrate priming by a S1P and the size-excluding role of the *Ec*RseP PDZ domain. In contrast, the processing of pro-σ^K^ by SpoIVFB, which does not contain a PDZ domain, occurs independently of a S1P. BofA and SpoIVFA, two regulatory membrane proteins, form a complex with SpoIVFB in the outer forespore membrane (Rudner and Losick 2002, Zhou and Kroos 2004). The formation of the complex is suggested to allow BofA to interact directly with the SpoIVFB active site, thereby inhibiting Pro-σ^K^ processing (Olenic et al. 2022a, Zhou and Kroos 2004). The serine proteases SpoIVB and CtpB are suggested to sequentially cleave the BofA: SpoIVFA complex, thereby liberating the active SpoIVFB to cleave pro-σ^K^ (Dong and Cutting 2003, Zhou and Kroos 2005, Campo and Rudner 2006). Importantly, transcription of serine protease is only activated in the forespore after it has been completely engulfed (Campo and Rudner 2006, Tan and Ramamurthi 2014), thereby ensuring that σ^K^ will be released into the mother cell to activate genes at the onset of the final sporulation stage. In this sense, SpoIVB acts as the primary mediator of signaling from the forespore to σ^K^ activation in the mother cell.

The SpoIVFB-mediated cleavage of pro-σ^K^ differs from the RIP-cascade paradigm in *E. coli* in multiple aspects. Most notably, SpoIVFB directly cleaves the transcription factor, not an anti-sigma factor, to produce the active form of σ^K^. Moreover, SpoIVFB mediated processing of pro-σ^K^ appears to be independent of canonical S1P cleavage, although proteolytic cleavage of the BofA: SpoIVFA complex is needed for SpoIVFB activation (Fig. 2B).

## S2P-mediated lysozyme resistance in Gram-positive bacteria

Lysozyme is a key component of the innate immune response. Lysozyme acts as a hydrolase, degrading the glycosidic bonds between N-acetylmuramic acid (NAM) and N-acetylglucosamide (NAG) in peptidoglycan in the bacterial cell wall, resulting in increased cell wall permeability and ultimately cell death (Ragland and Criss 2017). Several bacteria have developed sophisticated mechanisms to counteract the effect of lysozyme (Bera et al. 2005, Ho et al. 2011, Varahan et al. 2013, Ragland and Criss 2017, Ho and Ellermeier 2019, Pannullo and Ellermeier 2022).

In *B. subtilis*, lysozyme resistance is mediated through S2P-dependent activation of the ECF sigma factor σ^V^ (Ho et al. 2011, Hastie et al. 2013). In the absence of lysozyme, σ^V^ activity is hindered by the anti-sigma factor RisV (Yoshimura et al. 2004, Hastie et al. 2013). In the presence of lysozyme, an extracellular domain of RisV directly binds lysozyme resulting in conformational changes within the anti-sigma factor, thereby initiating the RIP-cascade (Hastie et al. 2014, Hastie et al. 2016). The signal peptidases SipS and SipT are suggested to cleave the extracellular region of RisV (Hastie et al. 2014, Castro et al. 2018). Following this primary cleavage, the S2P RasP (regulator of sigma protease, *Bs*RasP) cleaves RisV, thereby releasing the cytoplasmic region of RisV containing σ^V^ (Hastie et al. 2013). It is presumed that cytosolic proteases further process RisV, however, such proteases have not yet been identified. Once released into the cytosol, σ^V^ activates genes involved in lysozyme resistance, including *oatA* and *dltA*. OatA and DltA modify cell wall properties and charge through O-acetylation of the peptidoglycan and D-alanylation of teichoic acids, respectively (Guariglia-Oropeza and Helmann 2011, Ho et al. 2011).

A similar cascade is observed in the opportunistic pathogens *C. difficile* and *E. faecalis*. Deletion of RasP (*Cd*RasP) and RseP (*Efs*RseP, also known as Eep) the S2Ps of *C. difficile* and *E. faecalis*, respectively, results in decreased σ^V^ activity and lysozyme resistance (Varahan et al. 2013, Pannullo and Ellermeier 2022, Rouchon et al. 2022). In *C. difficile, Cd*RasP mediates σ^V^ activation by cleaving RsiV in response to lysozyme in a dose-dependent manner (Pannullo and Ellermeier 2022). *C. difficile* σ^V^ regulates the expression of several genes in response to lysozyme, including the peptidoglycan deacetylase PdaV, which removes acetyl groups from peptidoglycan in the cell wall (Ho et al. 2014, Woods et al. 2016, Kaus et al. 2020, Parthasarathy et al. 2021). In this bacterium, deacetylation of the peptidoglycan results in reduced affinity for lysozyme, thereby increasing lysozyme resistance. Interestingly, deletion of *pd*aV results in only a modest increase in lysozyme susceptibility. However, the combined loss of *pdaV* and another deacetylase, *pgdA*, resulted in a 1 000-fold reduction in lysozyme resistance, suggesting that these two deacetylases are redundant (Kaus et al. 2020). In a similar manner, σ^V^ of *E. faecalis* regulates expression of peptidoglycan deacetylase PgdA (Le Jeune et al. 2010, Benachour et al. 2012, Varahan et al. 2013, Parthasarathy et al. 2021), but also here, deletion of *pgdA* alone does not affect lysozyme resistance (Hébert et al. 2007, Benachour et al. 2012, Varahan et al. 2013, Parthasarathy et al. 2021). It is possible that a similar redundancy as observed in *C. difficile*, is found in *E. faecalis*, or that other σ^V^-dependent genes also contribute to the decrease in lysozyme resistance observed for the *E. faecalis* strains depleted of RseP and σ^V^.

Thus, the S2P-mediated activation of σ^V^ dependent lysozyme resistance appears highly conserved in *B. subtilis, C. difficile*, and *E. faecalis*. To date, no S1P has been identified for RsiV in *E. faecalis* or *C. difficile*. However, a signal peptidase cleavage site has been identified in RsiV for both species, suggesting that the S1P is a so far unknown signal peptidase (Hastie et al. 2014, Pannullo and Ellermeier 2022). In *E. faecalis*, σ^V^ is also suggested to contribute to a general stress response, as Δ*rseP* and Δ*sigV* significantly attenuated survival in response to multiple stressors, including heat, acid, and ethanol (Benachour et al. 2005, Varahan et al. 2013). Similar observations have been made in *Enterococcus faecium*, where mutations within *rseP* results in a 6–8-fold increase in lysozyme susceptibly as well as a reduction in desiccation tolerance (Reinseth et al. 2021). A more general role in stress response has so far not been observed for RasP and σ^V^ in *B. subtilis or C. difficile*.

## RseP and iron acquisition in Gram-negative bacteria

Iron acquisition is a key factor for colonization and infection of several pathogens (Sheldon et al. 2016, Llamas and Sánchez-Jiménez 2022). For pathogenic bacteria, iron resources in the environment are limited, as most of the host iron is present in high affinity iron binding complexes, such as hemoglobin and transferrin. To circumvent these limitations, bacteria have developed several sophisticated iron acquisition systems, including the production of siderophores and heme utilization (Sheldon et al. 2016, Llamas and Sánchez-Jiménez 2022). To ensure rapid adaption to changing environment, iron acquisition is regulated by ECF sigma factors in several Gram-negative bacteria.

In *P. aeruginosa*, an opportunistic human pathogen causing a range of different infections, particularly in immunocompromised and cystic fibrosis patients, the involvement of ECF sigma factors in iron acquisition has been extensively reviewed (Llamas et al. 2014, Chevalier et al. 2019, Llamas and Sánchez-Jiménez 2022). Interestingly, the S2P RseP, also known as MucP, is required for complete proteolysis of the anti-sigma factor and thus ECF sigma factor activity, in at least five pathways related to iron acquisition in *P. aeruginosa*: The pyoverdine, ferrichrome and ferrioxamine siderophore pathways, and the Has and Hxu heme pathways (Draper et al. 2011, Bastiaansen et al. 2014, Bastiaansen et al. 2015, Otero‐Asman et al. 2019). S2P mediated iron acquisition appears to be conserved in *Pseudomonas* species, as the deletion of *rseP* additionally eradicates the activity of multiple iron acquisition pathways in *P. putida* (Bastiaansen et al. 2014). Interestingly, several of the RseP-regulated iron acquisition pathways have also been implied in the pathogenicity of *P. aeruginosa* (Damron et al. 2016, Minandri et al. 2016, Cai et al. 2022). Most notably, the deletion of the sigma factor PvdS, which regulates pyoverdine production in a RseP-dependent manner, resulted in attenuated virulence in mouse lung infection (Wilderman et al. 2001, Draper et al. 2011, Imperi et al. 2013). In addition to pyoverdine production, σ^PvdS^ regulated the expression of the major virulence factors exotoxin A and PrpL, thereby having a dual role in *P. aeruginosa* virulence (Wilderman et al. 2001, Hunt et al. 2002, Ochsner et al. 2002, Minandri et al. 2016). The role of S2P and the RIP-cascade in *P. aeruginosa* virulence will be further discussed below.

S2P-mediated iron acquisition has also been implied in other gram-negative species. In *B. bronchiseptica*, a pathogen colonizing the respiratory tract of animals and humans, the S2P HurP (heme utilization receptor protease) is essential for heme utilization through the expression of the outer membrane receptor BhuR (King-Lyons et al. 2007). The complete regulation mechanism has not been fully elucidated; however, it is suggested that HurP cleaves the anti-sigma factor HurR in a heme-dependent manner. The modification of HurR is proposed to release the sigma factor HurI, which subsequently regulates the cellular response to the iron depleted environment and heme uptake. The S1P of this proposed RIP cascade has not yet been identified (King-Lyons et al. 2007). Curiously, *hurP* complements mutations in the S2P *rseP* in *V. cholerae* while *rseP* from *E. coli* complements *hurP* deletions in *B. bronchiseptica* (King-Lyons et al. 2007), indicating that substrate recognition and processing must be highly conserved in these species. To date, RseP has not been implied in iron acquisition in *V. cholerae*, however, *Ec*RseP was recently found to activate the ECF sigma factor FecI in response to ferric-citrate (Yokoyama et al. 2021).

## RasP and resistance to antimicrobials in *Bacillus* spp.

Several bacteria utilize alternative sigma factors to regulate genes in response to stressors such as antibiotics and antimicrobial peptides (Woods and McBride 2017). In *B. thuringiensis*, a Gram-positive bacterium commonly found in the soil, resistances to selected β-lactams are controlled by the ECF sigma factor σ^P^ (Fig. 2C). Upon activation, σ^P^ regulates expression of multiple β-lactamases and penicillin binding proteins (PBP) (Ho et al. 2022), providing *B. thuringiensis* with an arsenal of protection strategies. The activity of σ^P^ is hindered by the anti-sigma factor RsiP, which, in response to selected β-lactams, is successively cleaved by the S1P SipP and the S2P RasP (Ho et al. 2019, Nauta et al. 2022). It is suggested that penicillin-binding protein P (PbpP) acts as a sensor for β-lactams, somehow activating the S1P cleavage of the RIP cascade (Nauta et al. 2021). Δ*pbpP*, Δ*sipP*, and Δ*rasP* increase susceptibility to ampicillin and cefoxitin (Ho et al. 2019, Nauta et al. 2021, Nauta et al. 2022), underpinning the RIP cascade's role in σ^P^ mediated β-lactam resistance. Similar cascades is observed in *B. subtilis*, where σ^W^ participates in response to a variety of cell wall inhibiting antibiotics and antimicrobial peptides (Cao et al. 2002, Pietiäinen et al. 2005, Butcher and Helmann 2006). Activation of σ^W^ depends on the RIP-mediated cleavage of the anti-sigma factor RsiW by the S1P PrsW and the S2P RasP (Schöbel et al. 2004, Ellermeier and Losick 2006, Heinrich and Wiegert 2006). Although primarily considered as an antibiosis regulon, the *B. subtilis* σ^W^ responds to various stressors compromising cell wall integrity (Petersohn et al. 2001, Schöbel et al. 2004, Pietiäinen et al. 2005, Butcher and Helmann 2006, Ellermeier and Losick 2006). In contrast, *B. thuringiensis* σ^P^ is activated only by a specific subset of β-lactams, not other cell wall-targeting antibiotics, suggesting that σ^P^ is not activated in response to general cell wall stress (Ho et al. 2019). It should be noted that σ^P^ also has been linked to resistance against β-lactams in *Bacillus cereus* and *Bacillus anthracis* (Ross et al. 2009, Nauta et al. 2022). RasP is conserved in these closely related human pathogens, suggesting a role of RIP-mediated antibiotic resistance in these species. However, the role of RIP-mediated resistance to antibiotics in *B. cereus* and *B. anthracis* has not been extensively studied.

In this context, it is also interesting to note that a variation to the canonical RIP cascade involving the S2P RasP in *B. subtilis* was recently reported (Brunet et al. 2022). In this system, which is involved in sensing cell wall homeostasis, the anti-sigma factor RsgI is constitutively cleaved by a S1P. However, the two cleavage products remain associated as long as the cell wall is intact, thereby hindering site-2 cleavage by RasP. When a cell wall defect occurs, this is sensed by the extracellular domain on the cleaved RsgI, resulting in release of the cleavage product, followed by site-2 cleavage by RasP to release active σ^I^. This sigma factor activates genes needed to repair cell wall defects and has also been linked to changes in sensitivity to β-lactams (Patel et al. 2020, Brunet et al. 2022).

## The role of S2P in bacterial pathogenesis

As discussed above, S2P-mediated activation of ECF sigma factors plays a key role in the quick adaptation to extracytoplasmic stimuli in many bacteria. Rapid adaptation to changing environments is crucial for establishing infections, however S2P-mediated RIP have also been implied in the direct activation of virulence related genes (Fig. 2, Table 1). In the following section the role of S2P in prominent human pathogens will be discussed.

## MucP and mucoid production in *P. aeruginosa*

*Pseudomonas aeruginosa* is an opportunistic pathogen causing a wide range of nosocomial infections. Most notably, *P. aeruginosa* is a major cause of morbidity among patients with cystic fibrosis. Clinical *P. aeruginosa* isolated from patients with cystic fibrosis generally exhibit mucoid phenotype due to overproduction of alginate (Pritt et al. 2007, Hogardt and Heesemann 2010). It is suggested that alginate production contributes to protecting the bacteria against various stressors, including antibiotics and oxidative stress, thereby promoting persistence in the lungs and thereby virulence. In *P. aeruginosa*, genes involved in alginate production is controlled by the ECF sigma factor σ^22^, also known as σ^AlgT^ or σ^AlgU^ (Wood et al. 2006, Wood and Ohman 2009). Activation of σ^22^ is regulated in a RIP-depended manner in response to envelope stress, highly resembling the RseP-mediated activation of the extracytoplasmic stress response in *E. coli* (Fig. 2D) (Wood et al. 2006, Wood and Ohman 2009, Damron and Goldberg 2012). It has indeed been suggested that alginate production plays a part in a general stress response, as σ^22^ also regulates genes involved in protection against extracytoplasmic stress (Wood et al. 2006, Wood and Ohman 2009).

Under non-stress conditions, the anti-sigma factor MucA sequesters σ^22^ in the membrane, thereby inactivating the sigma factor (Li et al. 2019) (Fig. 2D). The PDZ domain of *P aeruginosa* DegS (*Pa*DegS), also known as AlgW, is suggested to recognize C-terminal residues of the envelope protein MucE and possibly other OMP, which are accumulated under cell wall stress (Qiu et al. 2007, Cezairliyan and Sauer 2009). This interaction initiates *Pa*DegS mediated cleavage of MucA, which is proposed to subsequently trigger a secondary cleavage by *Pa*RseP (Qiu et al. 2007, Cezairliyan and Sauer 2009, Wood and Ohman 2009, Damron and Hongwei 2011, Damron and Goldberg 2012). MucA is suggested to be further processed by proteases such as ClpXP in the cytosol, finally resulting in the release of activated σ^22^ (Qiu et al. 2008). Under non-stress conditions, MucB, the equivalent to RseB in *E. coli*, binds to MucA, thereby inhibiting RIP-mediated cleavage of the anti-sigma factor (Fig. 2A and D) (Cezairliyan and Sauer 2009, Wood and Ohman 2009, Li et al. 2019).

It is interesting to note that clinical *Pseudomonas* isolates from cystic fibrosis patients often contain mutation within MucA, commonly resulting in a C-terminal truncation of the anti-sigma factor and subsequent increased σ^22^ activity (Bragonzi et al. 2006, Pulcrano et al. 2012). In *E. coli*, truncation of RseA results in *Ec*RseP cutting of RseA independent of site-1 cleavage (Li et al. 2009, Hizukuri et al. 2014). A similar mechanism is proposed for the cleavage of truncated MucA in *P. aeruginosa*, explaining the constitutive mucoid phenotype commonly observed for MucA mutants (Damron and Goldberg 2012). As discussed above, RseP of *P. aeruginosa* has also been implicated in iron-uptake and regulation of virulence determinators exotoxin A and PrpL. The combined role of RseP-mediated regulation of iron uptake, alginate production and expression of virulence enhancing genes, makes it tempting to speculate that a *rseP* deletion would have a striking effect on *P. aeruginosa* pathogenicity. However, the role of pseudomonal RseP has not yet been investigated in infection models *in vivo*.

## Rip1 and virulence in *M. tuberculosis*

*Mycobacterium tuberculosis* is an obligate human pathogen and a leading cause of infection worldwide (Antimicrobial-Resistance-Collaborators 2022). The complex composition of the *M. tuberculosis* cell envelope makes these infections difficult to treat and is known to play a key role in the persistence and virulence of this pathogen (Garcia-Vilanova et al. 2019, Maitra et al. 2019). Transcriptional analysis in the presence or absence of detergents revealed that the *M. tuberculosis* S2P Rip1 regulates genes involved in lipid metabolism in response to a changing environment (Makinoshima and Glickman 2005). In line with this evidence, Δ*rip1* displays altered cell morphology and loss of ability to cord (Makinoshima and Glickman 2005), which is an established virulence trait relying on a glycolipid known as the cord-factor in the mycobacterial cell-envelope. When challenged in an aerosol murine model, Δ*rip1* showed impaired growth during both the acute and chronic phase of infection (Makinoshima and Glickman 2005, Schneider et al. 2014, Buglino et al. 2021). Specifically, by 22 weeks, the bacterial titers from the lungs were 10 000-fold lower for Δ*rip1* compared to wild-type, while Δ*rip1* was completely abolished from the liver (Makinoshima and Glickman 2005), and by 43 weeks, Δ*rip1* was completely cleared from the lungs, showing no detectable colony forming units (CFU) in the majority of the mice (Schneider et al. 2014). The deletion phenotype was rescued when complemented with the wild-type Rip1, showing that attenuated virulence is Rip1 dependent (Makinoshima and Glickman 2005).

Although the severe attenuation of *M. tuberculosis* virulence caused by the single gene deletion of *rip1* is striking, the mechanism of Rip1 in *M. tuberculosis* virulence remains somewhat elusive. In fact, Rip1 cuts four independent anti-sigma factors controlling the activity of σ^K^, σ^L^, σ^M^ and σ^D^ (Sklar et al. 2010, Schneider et al. 2014). Interestingly, Δ*sigKLM* does not attenuate bacterial growth in aerosol murine model, suggesting that the Rip1-dependent virulence observed for Δ*rip1* is independent of these pathways (Schneider et al. 2014). This points to a key role of σ^D^ in the virulence mechanism, which notably controls expression of multiple virulence factors, including *fbpA* and the resuscitation promoting factor RpfC (Raman et al. 2004, Calamita et al. 2005). Thus, it was surprising to note that single deletion Δ*sigD* only caused modest decrease in virulence compared to the ΔRip1 (Raman et al. 2004, Calamita et al. 2005). So far, the effect of the quadruple mutant (Δ*sigKLMD*) has not been investigated *in vivo*.

Adding to the complexity of Rip1s role in virulence, a recent study revealed that Rip1 contributes to defense against host-imposed stressors (Buglino et al. 2021). When challenging Δ*rip1* with various stressors, Rip1 was found to be essential for protection against metal and nitrosative stress. Most importantly, the observed attenuated growth of Δ*rip1* in acute infection in mice was reversed in the absence of nitric oxide production, suggesting that Rip1 directly contributes to pathways defending *M. tuberculosis* against host-produced nitric oxide (Buglino et al. 2021). Interestingly, no Rip1-dependent defects were observed when challenging the *Δrip1* mutant with starvation, lysozyme, iron deficiency or an acidic environment (Buglino et al. 2021), as previously observed for other S2P mutants (as discussed above).

## RseP and pheromone production in *E. faecalis*

S2P was generally believed only to regulate transcription factor activity. However non-anti-sigma factor substrates have been identified for S2P in multiple species, with *E. faecalis* being the earliest example (Chen et al. 2005, Matson and DiRita 2005, Mukherjee et al. 2009, Saito et al. 2011, Schilcher et al. 2020). Although considered commensal enterococci have emerged as important healthcare-associated pathogens in the last decades, causing a wide range of infections, including urinary tract infections (UTIs), endocarditis, and bacteremia (Cattoir and Leclercq 2013, Reinseth et al. 2019). The S2P of *E. faecalis, Efs*RseP, also known as Eep (enhanced expression of pheromone), increases the production of multiple sex pheromones, including cCF10, cAD1 and cPD1 (An et al. 1999, An and Clewell 2002, Chandler et al. 2005, Chandler and Dunny 2008), which are critical for conjugation of plasmids between bacterial cells. A model has been proposed in which lipoprotein precursor is sequentially digested by a type II signaling peptidase and *Efs*RseP, before a ABC transporter actively transport the resulting mature pheromones outside the cell (Varahan et al. 2014) (Fig. 2E). S2P involvement in pheromone maturation has also been suggested in several species of *Streptococcus, L. monocytogenes*, and *S. aureus*, indicating that S2P-mediated pheromone processing may be a conserved mechanism among some gram-positive bacteria (Denham et al. 2008, Chang et al. 2011, Pérez-Pascual et al. 2015, Xayarath et al. 2015, Cheng et al. 2020, Schilcher et al. 2020).

In addition to its role in pheromone processing, *Efs*RseP has been suggested as a key factor in *E. faecalis* virulence (Frank et al. 2012, Frank et al. 2013). When challenged in a rabbit endocarditis model, Δ*rseP* was severely attenuated, showing a 4-log_10_ decrease in CFU recovered from the heart valves compared to the wild-type. The attenuation defects observed for Δ*rseP* could be rescued by *in trans* expression of *rseP* (Frank et al. 2012). Moreover, Δ*rseP* resulted in a reduced bacterial load recovered from the kidneys in a murine catheter-associated UTI model (Frank et al. 2013). It should be noted that the defects in virulence observed for the Δ*rseP* is likely not attributed to loss of plasmid conjugation, as both *in vivo* studies were performed using *E. faecalis* OG1RF—a well characterized laboratory strain which almost completely lacks mobile genetic elements (Bourgogne et al. 2008). *Efs*RseP dependent virulence is therefore most likely not related to lack of sex pheromones maturation. On the other hand, σ^V^ may be involved. As discussed above, *Efs*RseP induces lysozyme resistance through RIP-mediated activation of the ECF σ^V^ (Varahan et al. 2013). Indeed, Δ*sigV* attenuated *E. faecalis* virulence in a murine UTI and systemic infection (Le Jeune et al. 2010). This indicates that, at least to some extent, reduction in σ^V^ activation may contribute to the reduced virulence observed for strains deficient in *Efs*RseP. Nevertheless, the exact mechanisms underlying *Efs*RseP-mediated virulence remains to be confirmed.

## Eep and virulence in *S. aureus*

*S. aureus* is a major human pathogen causing a wide range of both nosocomial and community acquired infections. Small linear peptides are suggesting a key role in multiple cellular processes in Gram-positive bacteria, including signaling, competence development, and virulence (Håvarstein et al. 1995, Thoendel and Horswill 2010, Chang et al. 2011, Xayarath et al. 2015). Similar to what was discussed above for *E. faecalis*, the *S. aureus* S2P (*Sa*Eep) is involved in maturation of multiple small linear peptides, including the sex pheromone cAM373 (Cheng et al. 2020, Schilcher et al. 2020). A proteomic analysis showed that Δ*eep* in *S. aureus* affected the levels of more than 55 proteins, including a significant decrease of several proteins involved in bacterial adhesion (i.e. SpA, SasG and FnbA) (Cheng et al. 2020). Indeed, Δ*eep* showed reduced adhesion to human epithelial cells two hours post-infection. Overexpression of Spa, SasG, and FnbA increased adhesion in the Δ*eep* strain, suggesting that the reduced ability to adhere is directly linked to reduced production of important adhesion proteins in the mutant (Cheng et al. 2020). Furthermore, when challenging a murine blood infection model, Δ*eep* showed significantly decreased virulence, and survival (Cheng et al. 2020). In this study, CFU counts retrieved from the liver, but not the kidneys, could be restored to wild-type levels when SpA was overexpressed in the Δ*eep* mutant, suggesting that *Sa*Eep may affect other aspects of infection besides adhesion (Cheng et al. 2020).

The ECF factor σ^S^ has been suggested to play a role in *S. aureus* virulence (Shaw et al. 2008, Miller et al. 2012). Interestingly, a *S. aureus* homolog of the S1P PrsW in *B. subtilis* is suggested to be involved in σ^S^ activation (Krute et al. 2015). These observations, taken together with the fact that *Sa*Eep controls the expression of a vast range of proteins, make it tempting to speculate that *Sa*Eep and RIP-mediated activation of ECF factors play roles in *S. aureus* virulence. However, the potential role of Eep in *S. aureus* ECF factor activity remains to be elucidated.

## RseP and virulence regulation in *V. cholerae*

As reviewed above, RIP is a common mechanism for activating transcriptional factors by releasing them from the membrane. In contrast, TcpP, a transcription factor needed for activation of the master virulence regulator ToxT in *V. cholerae*, is active when bound to the membrane but inactivated following proteolysis (Fig. 2F). ToxT directly activates multiple virulence associated genes in *V. cholerae*, including genes encoding cholerae toxin and the toxin-co-regulated pilus (Matson et al. 2007). Under non-virulence inducing conditions, TcpP is degraded in a RIP-dependent manner, thereby negatively regulating the expression of *toxT* and downstream virulence associated genes (Matson and DiRita 2005, Teoh et al. 2015). In this process, the periplasmic protein Tsp (tail-specific protease) performs the primary cleavage of TcpP, while the S2P cleavage is performed by *V. cholerae* RseP (*Vc*RseP, also known as Yael) (Matson and DiRita 2005, Teoh et al. 2015). During virulence promoting conditions, another protein known as TcpHis suggested to interact with TcpP, thereby protecting it from RIP mediated cleavage, thus allowing *toxT* expression (Beck et al. 2004, Matson and DiRita 2005).

Another level of regulatory complexity in this system is added by another membrane-bound transcription factor known as ToxR and its effector protein ToxS (Fig. 2E). ToxR is thought to additionally enhance the TcpP mediated activation of *toxT* (Fig. 2E) (Almagro-Moreno et al. 2015b, Krukonis et al. 2000). Notably, *V*cRseP has been implied in ToxR cleavage under nutrient limitation at alkaline pH (Almagro-Moreno et al. 2015a). ToxR cleavage has been associated with the entry of *V. cholerae* to a dormant state, suggesting that RIP-mediated inactivation of virulence regulators TcpP/ToxR and entry into a nonculturable state are linked (Almagro-Moreno et al. 2015a).

## Targeting S2P in the context of antimicrobial therapy

Despite the evident role of S2P in bacterial physiology and virulence, only a limited number of inhibitors have been shown to target S2P. Several challenges hamper the development of specific S2P inhibitors. Most notably, S2P are conserved among all kingdoms of life (Kinch et al. 2006), making adverse interaction between an inhibitor and the host S2P a potential concern. Increased biochemical and structural understanding of S2P may contribute to developing specific inhibitors with minimal off-target effects. Novel protease inhibitors are generally rationally designed based on the known structure of the protease itself, or by mimicking the interaction with known protease substrates and/or inhibitors. To date, only three S2P has been fully structurally characterized: *Ec*RseP, RseP of *Kangiella koreensis* and the S2P of *M. jannaschii* (Feng et al. 2007, Imaizumi et al. 2022). Despite these challenges, S2P remains an attractive drug target, with a limited number of inhibitors been identified.

Most notably, a subgroup of bacteriocins known as the LsbB family, target the S2P of selected gram-positive bacteria (Uzelac et al. 2013, Ovchinnikov et al. 2014, Ovchinnikov et al. 2017, Kranjec et al. 2021). Bacteriocins are small, ribosomally synthesized peptides produced by bacteria that inhibit the growth of other bacteria in competition for nutrients and ecological niches. These antimicrobial peptides have predominantly been exploited as food preservatives; however, bacteriocins are receiving increased scientific interest as promising alternatives and/or complements to antibiotics due to their potent activity against multiple human pathogens (Cotter et al. 2013, Soltani et al. 2021). Among the members of the LsbB family, enterocin EJ97 (EntEJ97) and enterocin K1 (EntK1) show the greatest therapeutic potential. EntEJ97 and EntK1 show potent activity against vancomycin-resistant enterococci (VRE) at nanomolar concentrations *in vitro* (Ovchinnikov et al. 2017, Reinseth et al. 2021). Notably, EntEJ97 and EntK1 are autoclavable and retain antimicrobial activity in blood, highlighting their therapeutic potential against systemic infections (Reinseth et al. 2021). The potent activity of EntK1 and EntEJ97 have been attributed to a specific interaction with the enterococcal S2P (Ovchinnikov et al. 2017, Kristensen et al. 2022). As discussed above, enterococcal RseP is suggested to be important for stress tolerance and virulence in enterococci (Benachour et al. 2005, Frank et al. 2012, Frank et al. 2013, Varahan et al. 2013, Reinseth et al. 2021). Indeed, while spontaneous EntK1- and EntEJ97 resistant mutants can emerge due to mutations in *rseP*, these mutants show a 6–8-fold increase in lysozyme susceptibility, reduced tolerance to heat and a significant reduction in desiccation tolerance (Ovchinnikov et al. 2017, Reinseth et al. 2021). Thus, resistance development seems to come at a high cost for the bacteria cell.

EntK1 and EntEJ97 are particularly attractive bacteriocins, not only because they act on VRE, but also because they are synthesized without a N-terminal leader sequence and contain no post-translational modifications (Ovchinnikov et al. 2017, Kristensen et al. 2022). This makes EntK1 and EntEJ97 an ideal starting point for bioengineering of novel S2P targeting antimicrobials. Indeed, the construction of a EntK1/EntEJ97 hybrid bacteriocin, designated Hybrid 1, exhibited a novel inhibition spectrum and improved activity compared to the parental bacteriocins (Kranjec et al. 2021). It was recently shown that the antimicrobial activity of EntK1 is dependent on its interactions with the PDZ-domain and Asn359 in the extended LDG motif of RseP in *E. faecium* (Kristensen et al. 2022). Interestingly, these regions have been implicated in substrate interaction for other bacterial S2P (e.g *Ec*RseP, *B. subtilis* SpoIVFB) (Olenic et al. 2022b, Koide et al. 2008, Zhang et al. 2013, Akiyama et al. 2015, Akiyama et al. 2017), and are conserved among several of the human pathogens discussed in this review. While these bacteriocins hold significant potential as novel antimicrobials targeting S2P, the *in vivo* efficacy and potential cytotoxicity of these peptides remain to be investigated in detail.

Besides antimicrobial peptides, two commercially available protease inhibitors have been shown to have a surprising effect on S2P activity. Nelfinavir (Viacept), an FDA-approved protease inhibitor used in HIV therapy, is suggested to inhibit human S2P. The drug results in increased accumulation of the human S2P substrates AFT6 and SREBP1 (Guan et al. 2011, Guan et al. 2015). In humans, S1P and S2P are involved in lipid metabolism and unfolded protein response (endoplasmatic reticulum stress) (Danyukova et al. 2021). Interestingly, nelfinavir shows promising anti-cancer properties, and the therapeutic potential of nelfinavir is currently being investigated for several types of cancers (Gills et al. 2007, Gantt et al. 2013, Guan et al. 2015, Kawabata et al. 2021). Nelfinavir and nelfinavir-analogs inhibit the proteolytic activity of *Mj*S2P, the S2P in *M. jannaschii, in vitro*, indicating that this drug may be a potent inhibitor of bacterial S2P (Guan et al. 2015). However, the antimicrobial potential of nelfinavir is largely unexplored.

Additionally, Konovalova and colleagues recently identified batimastat, a broad-spectrum protease inhibitor known to target eukaryotic metalloproteases, as a potent inhibitor of *Ec*RseP (Konovalova et al. 2018). Structural analysis of the *Ec*RseP in complex with batimastat revealed that the binding mode of batimastat to *Ec*RseP depends on residues and regions known to be crucial for interaction with the native substrate RseA (Fig. 1A) (Imaizumi et al. 2022). Notably, as also seen for RseP: EntK1 interaction, this includes the extended LDG domain (Imaizumi et al. 2022, Kristensen et al. 2022). However, batimastat was identified as *Ec*RseP inhibitor in a screen using an efflux-deficient (Δ*tolC*) strain, and batimastat have no inhibitory effect on the wild-type efflux-intact strains (Konovalova et al. 2018). This likely hampers the therapeutic potential of batimastat in antimicrobial infections.

## Future perspective and concluding remarks

S2P-mediated RIP is a well conserved signaling mechanism common to all forms of life. Notably, S2P and RIP play a key role in physiology and virulence in multiple human pathogens, including enterococci, staphylococci, and *M. tuberculosis*. The recent advances in the field have increased our understanding of the various molecular mechanisms underlying S2P-mediated signaling and has raised the exciting possibility that S2P may serve as novel antimicrobial targets. So far, the antimicrobial peptides of the LsbB bacteriocin family are the only known antimicrobials specifically targeting S2P; however, as our structural and biochemical insights into S2P increase, more inhibitors are likely to be developed. Tools like Alphafold and artificial intelligence (AI) are revolutionizing the field of drug discovery by providing new ways to predict protein structures and identify potential inhibitors. The use of these technologies is expected to grow in the future as they will help researchers to understand the structure of S2P better and screen large libraries of potential inhibitors for those that effectively bind and inhibit S2P.
